# Recombinant Duck Enteritis Virus-Vectored Bivalent Vaccine Effectively Protects Against Duck Hepatitis A Virus Infection in Ducks

**DOI:** 10.3389/fmicb.2021.813010

**Published:** 2021-12-22

**Authors:** Fuchun Yang, Peng Liu, Xiaohan Li, Rui Liu, Li Gao, Hongyu Cui, Yanping Zhang, Changjun Liu, Xiaole Qi, Qing Pan, Aijing Liu, Xiaomei Wang, Yulong Gao, Kai Li

**Affiliations:** Avian Immunosuppressive Diseases Division, State Key Laboratory of Veterinary Biotechnology, Harbin Veterinary Research Institute, Chinese Academy of Agricultural Sciences, Harbin, China

**Keywords:** duck enteritis virus, duck hepatitis A virus, bivalent vaccine, P1 protein, 3C protein

## Abstract

Duck enteritis virus (DEV) and duck hepatitis A virus (DHAV) are prevalent duck pathogens, causing significant economic losses in the duck industry annually. Using a fosmid-based rescue system, we generated two DEV recombinants, rDEV-UL26/27-P13C and rDEV-US7/8-P13C, in which the P1 and 3C genes from DHAV type 3 (DHAV-3) were inserted into the DEV genome between genes UL26 and UL27 or genes US7 and US8. We inserted a self-cleaving 2A-element between P1 and 3C, allowing the production of both proteins from a single open reading frame. P1 and 3C were simultaneously expressed in infected chicken embryo fibroblasts, with no difference in growth kinetics between cells infected with the recombinant viruses and those infected with the parent DEV. Both recombinant viruses induced neutralizing antibodies against DHAV-3 and DEV in ducks. A single dose of the recombinant viruses induced solid protection against lethal DEV challenge and completely prevented DHAV-3 infection as early as 7 days post-vaccination. These recombinant P1- and 3C-expressing DEVs provide potential bivalent vaccines against DEV and DHAV-3 infection in ducks.

## Introduction

Ducks have many commercial uses, making them one of the most important waterfowl. However, duck production is threatened by many pathogens, of which the most important are duck hepatitis A virus (DHAV) and duck enteritis virus (DEV, a herpesvirus) (Yugo et al., 2016; Dhama et al., 2017). This is a major factor in China, where up to four billion ducks are reared annually for food and clothing (Liu et al., 2011a).

Duck hepatitis A virus belongs to the genus *Avihepatovirus* in the family Picornaviridae. It has a linear, single-stranded positive-sense RNA genome comprising a large open reading frame encoding a polyprotein, as well as untranslated regions (Wen et al., 2018). Its P1 region encodes the P1 protein, which is translated within this large open reading frame and is hydrolyzed into the structural capsid proteins VP0, VP1, and VP3 by the viral 3C protease (Kim et al., 2006; Wang et al., 2018). DHAV is the most common cause of duck virus hepatitis (DVH), which induces an acute, highly contagious, and rapidly progressing fatal disease in young ducklings, causing substantial economic losses in the duck industry worldwide (Wen et al., 2018). The DHAV strains are classified into three serotypes: the traditional serotype 1 (DHAV-1), distributed worldwide (Wang et al., 2008); serotype 2 (DHAV-2), endemic only in Taiwan (Tseng and Tsai, 2007); and the novel serotype 3 (DHAV-3). In Southeast Asia, DVH is caused mainly by DHAV-1 and DHAV-3 (Wen et al., 2018). DHAV-1 infection is currently controlled using modified live attenuated vaccines (Roh and Kang, 2018); in China, this has been officially approved for vaccinating breeder ducks since 2013. Although DHAV-3 accounts for an increasing proportion of DHAV infections in mainland China (Liu et al., 2011b), South Korea (Kim et al., 2007), and Vietnam (Roan et al., 2015), no commercial live vaccine suitable used in young ducklings is currently available. A safe and efficient vaccine for DHAV-3 is required.

Duck enteritis virus, also called duck plague virus, causes an acute contagious disease among Anseriformes (ducks, geese, and swans), resulting in high mortality and reduced egg production in domestic and wild waterfowl (Dhama et al., 2017). Attenuated DEV strains, including C-KCE and clone 03 from embryonated chicken eggs, have been routinely used as live vaccines in ducks for over half a century, without posing safety concerns for humans and domestic animals (Liu et al., 2011a; Sun et al., 2017). As with other herpesviruses, the large genome of DEV (approximately 158 kb) makes it highly suitable as a live viral vector for inserting and expressing the foreign antigen genes of other pathogens and for developing multivalent vaccines (Liu et al., 2011a; Chen et al., 2014; Zou et al., 2016; Sun et al., 2017).

Mixed DHAV and DEV infection has increasingly become frequent in domestic ducks, worsening the economic loss. Developing a bivalent vaccine that simultaneously acts against DHAV-3 and DEV is the most economical method for reducing losses in the duck industry. To address this, we generated two recombinant DEVs, with the P1 and 3C genes of a DHAV-3 virus inserted at different sites in the DEV vaccine strain genome via transfection of overlapping fosmid DNAs. The recombinant viruses were further evaluated *in vitro* and *in vivo* for antigen expression, replication, stability, and protective efficacy against DHAV-3 and DEV challenge in ducks.

## Materials and Methods

### Animals and Ethics Statement

The specific-pathogen-free (SPF) ducks, fertilized SPF chicken eggs, and duck eggs used in this study were purchased from the State Resource Center of Laboratory Animal for Poultry (Harbin, China). Ten-day-old SPF chicken and duck embryos were used to prepare primary chicken embryo fibroblasts (CEFs) and duck embryo fibroblasts (DEFs). This study was carried out according to the Guide for the Care and Use of Laboratory Animals of the Ministry of Science and Technology of China. The use of SPF eggs, embryos, and ducks and the animal experiments were approved by the Animal Ethics Committee of Harbin Veterinary Research Institute of the Chinese Academy of Agricultural Sciences and performed following animal ethics guidelines and approved protocols [SYXK (Hei) 2017-009].

### Viruses and Cells

The attenuated DEV vaccine strain C-KCE (Yang et al., 2013) was propagated in primary CEFs and used as the parent virus to produce the recombinant DEV. The virulent DEV CSC (Yang et al., 2014) and DHAV-3 A3 strains were propagated in 10-day-old SPF duck embryonated eggs and used as the challenge viruses.

### Construction of Fosmids With Insertion of DHAV-3 P1 and 3C Genes

**FIGURE 1:**
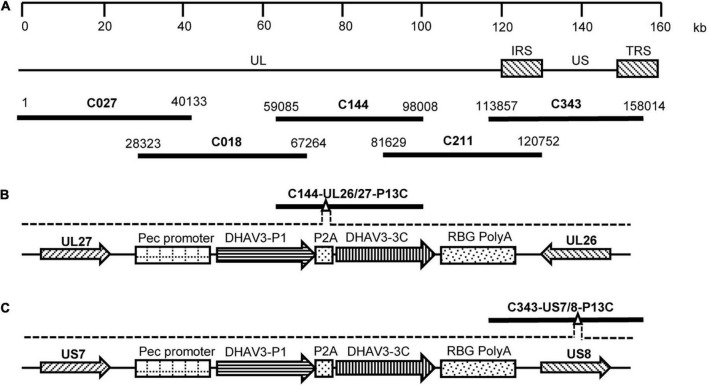
Construction of fosmids containing the P1 and 3C genes of DHAV-3. **(A)** The five fosmid combinations used for the generation of the DEV attenuated vaccine strain. Numbers represent the location of each fosmid fragment in the DEV vaccine strain genome. **(B)** The schematic diagrams of the recombinant fosmid with the P1 and 3C genes inserted between UL26 and UL27 in the DEV genome. **(C)** The schematic diagrams of the recombinant fosmid with the P1 and 3C genes inserted between US7 and US8 in the DEV genome.

Five fosmids (C027, C018, C144, C211, and C343), containing sequences spanning the entire genome of the DEV vaccine strain C-KCE (GenBank accession no. KF263690) were constructed during our preliminary studies and used for insertion of DHAV-3 P1 and 3C genes (Figure 1A). A dual-selection marker cassette containing the kanamycin resistance gene (KanR) and the ccdB gene flanked by the attR1 and attR2 sequences was first inserted into the gene junction between the UL26 and UL27 genes, or between the US7 and US8 genes, in the C144 or C343 fosmids, via Red/ET recombination using the counter-selection BAC modification kit (Gene Bridges GmbH, Heidelberg, Germany) to facilitate the insertion of foreign genes into the DEV genome. The modified fosmids containing the selection markers were designated C144-UL26/27-KanccdB and C343-US7/8-KanccdB, respectively.

The gene fragment P1-P2A-3C, containing the P1 and 3C genes of DHAV-3 and a self-cleaving 2A-element of porcine teschovirus-1 (Szymczak-Workman et al., 2012), was synthesized and cloned into the pCAGGS vector under the control of the CMV enhancer and β-actin promoter. The constructed P1-P2A-3C cassette was then used to replace the gus gene in the plasmid pENTR-gus (Invitrogen), and the resultant entry plasmid was designated pENTR-P13C. To insert the P1-P2A-3C cassette into the DEV genome, the entry plasmid pENTR-P13C was mixed with the modified fosmids C144-UL26/27-KanccdB and C343-US7/8-KanccdB and treated with LR Clonase II enzyme (Invitrogen). The mixtures were transformed into competent *Escherichia coli* EPI300-T1 cells. The resultant fosmids with P1-P2A-3C cassette insertion were designated C144-UL26/27-P13C (Figure 1B) and C343-US7/8-P13C (Figure 1C).

### Rescue of Recombinant Viruses From Overlapping Fosmid DNAs

The five fosmids with or without P1-P2A-3C insertion were used to rescue the recombinant viruses. Viral DNA inserts were released from the purified fosmids by digestion with *Sbf*I and were purified via phenol-chloroform extraction and ethanol precipitation before transfection. Two micrograms of each fosmid DNA were used to transfect the primary CEFs in 60 mm dishes, using the calcium phosphate procedure (Morgan et al., 1990). The cytopathic effect-positive samples were harvested and characterized via electron microscopy. To verify that the P1-P2A-3C cassette was inserted at the desired sites, viral genomic DNA was isolated from the rescued viruses and analyzed using PCR and sequencing.

### Confirmation of P1 and 3C Gene Expression

Expression of DHAV-3 P1 and 3C by recombinant DEVs was confirmed via indirect immunofluorescence assay. Briefly, CEFs in 6-well tissue culture plates were infected with the rescued viruses for 3 days. The medium was aspirated, and the cells were fixed with absolute ethanol for 20 min at room temperature. The fixed cells were incubated with rabbit anti-DHAV3-VP1 or rabbit anti-DHAV3-3C polyclonal antibodies in a wet box for 60 min at 37°C, then reacted with TRITC-labeled goat anti-rabbit IgG antibody (1:100 solution) (Sigma, St. Louis, MO, United States) in a wet box for 60 min at 37°C. After being washed five times, the cells were examined via fluorescence microscopy.

### Growth and Stability of the Recombinant Viruses

Chicken embryo fibroblasts cultured in 6-well plates were inoculated with different viruses at a multiplicity of infection of 0.01 and maintained at 37°C in 5% CO_2_ for 4 days to investigate the growth of the recombinant DEVs. The cells and supernatants were harvested at 24 h intervals, and the titer of infectious progeny present in the culture was determined as the median tissue culture infectious dose (TCID_50_) per mL, using the Reed-Muench formula (Reed and Muench, 1938). The mean ± standard deviation (SD) was calculated from three independent experiments. To evaluate the genetic stability of the foreign gene in the recombinant virus, we passaged the virus in the CEFs 20 times. The inserted genes were detected via PCR and sequencing, and P1 and 3C expression were confirmed using immunofluorescence assay as described.

### Protection Against DHAV-3 and Duck Enteritis Virus Challenge

All the animal experiments were performed using SPF ducks housed in filtered-air, negative-pressure isolation units. The ducks were given free access to food and water. To evaluate the protective efficacy of the recombinant viruses against challenge by the virulent DHAV-3 and DEV, each group of 20 ducks was inoculated subcutaneously with 1,000 times the 50% egg lethal dose (1000 ELD_50_) of the rescued viruses at 1 day of age. At 7 days post-vaccination, 10 ducks in each group were intramuscularly challenged with 100 ELD_50_ of the virulent DHAV-3 A3 strain, and the remaining 10 ducks were intramuscularly challenged with 1,000 minimum lethal doses of the virulent DEV CSC strain. Ten unvaccinated and unchallenged ducks were used as the healthy controls. The ducks were examined for clinical signs and mortality for 2 weeks after the challenge. The dead and surviving ducks were observed for gross lesions in the liver, spleen, kidneys, esophagus, intestine, thymus, and bursa.

### Serological Tests

Serum samples were collected from ducks 7 days post-vaccination and tested via virus neutralization assays. To determine the viral neutralizing antibody against DHAV-3, triplicates of heat-inactivated serum samples were serially diluted two-fold and mixed with an equal volume of 100 ELD_50_ of the virulent DHAV-3 A3 strain. After 1 h of incubation, the mixtures were added to 10-day-old embryonated SPF duck eggs, followed by further incubation for 7 days. The neutralizing antibody titer was determined as log_2_ of the reciprocal of the highest dilution at which there were no dead eggs.

To determine the viral neutralizing antibody against DEV, triplicates of heat-inactivated serum samples were serially diluted two-fold and mixed with an equal volume of 100 TCID_50_ of the virulent DEV CSC strain. After 1 h of incubation, the mixtures were added to monolayers of DEFs, followed by further incubation for 5 days. The neutralizing antibody titer was determined as log_2_ of the reciprocal of the highest dilution at which there were no visible cytopathic effects.

### Statistical Analysis

The results are presented as mean ± SD. One-way ANOVA was used to evaluate the statistical difference among groups, using SPSS 17.0 (SPSS Inc., Chicago, IL, United States). Statistical significance was set at *P* < 0.05 for all tests.

## Results

### Generation of Recombinant Duck Enteritis Viruses Expressing DHAV-3 P1 and 3C Genes

**FIGURE 2:**
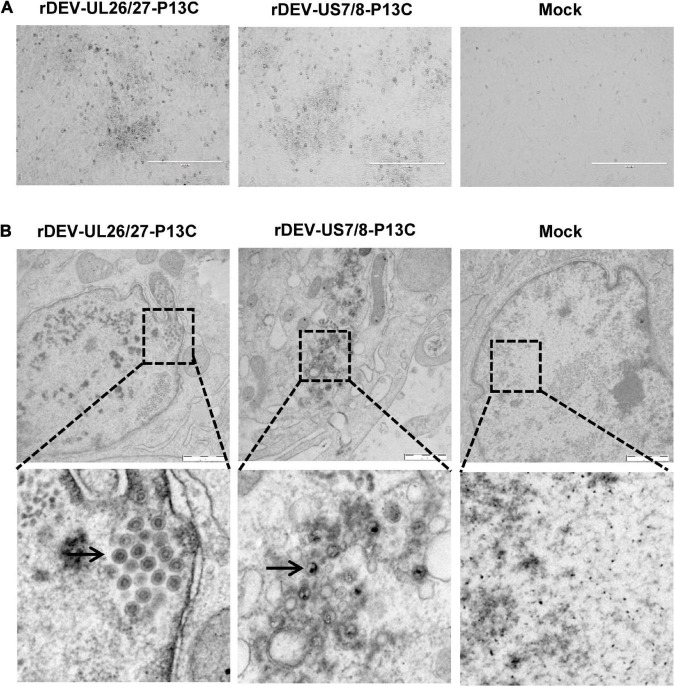
Generation of recombinant DEVs with the P1 and 3C genes insertion. **(A)** The cytopathic effects induced by the recombinant DEVs containing the DHAV-3 P1 and 3C genes in CEFs. **(B)** Electron microscopy detection of the recombinant DEVs in CEFs. Bar length, 1 μm. Arrows represent the DEV virions detected in infected cells.

**FIGURE 3:**
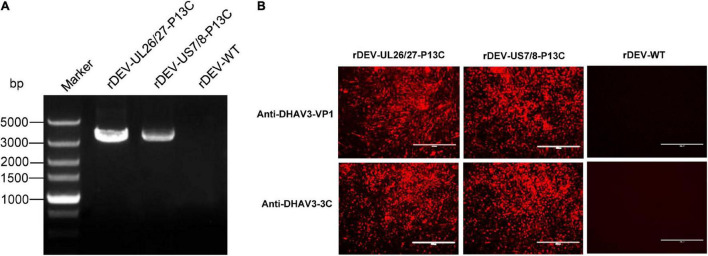
Detection of P1 and 3C expression by recombinant DEVs in infected CEFs. **(A)** PCR amplification of the P1-P2A-3C cassette from the recombinant viruses. **(B)** Detection of P1 and 3C expression by the recombinant viruses in indirect immunofluorescence assay with anti-DHAV3-VP1 and anti-DHAV3-3C antibodies.

The P1-P2A-3C cassette was inserted into the gene junction between UL26 and UL27, or between US7 and US8, in the DEV vaccine strain genome. For transfection, we used the modified fosmids, C144-UL26/27-P13C (Figure 1B) and C343-US7/8-P13C (Figure 1C), which replaced the parental fosmids, C144 and C343, respectively. After being blindly passaged once in CEFs, DEV-typical plaques appeared in the CEFs transfected with the DNA combinations (Figure 2A). Electron microscopy confirmed the successful rescue of the recombinant viruses (Figure 2B). Insertion of the P1-P2A-3C cassette at the proper site was confirmed by PCR and sequencing, using a forward primer specific to the P1 gene and a reverse primer matching the UL26 or US8 sequences (Figure 3A). The recombinant DEVs were designated rDEV-UL26/27-P13C, with the P1 and 3C genes inserted between UL26 and UL27, and rDEV-US7/8-P13C, with the P1 and 3C genes inserted between US7 and US8, in the DEV genome.

The expression of P1 and 3C proteins by the recombinant viruses was confirmed via indirect immunofluorescence assay. The cells infected with rDEV-UL26/27-P13C and rDEV-US7/8-P13C reacted with both the anti-DHAV3-VP1 and anti-DHAV3-3C antibodies, emitting a red fluorescent signal, whereas the parental virus-infected cells did not react with the antibodies (Figure 3B). These results indicate that recombinant DEVs co-expressing DHAV-3 P1 and 3C genes were successfully generated.

### Growth Kinetics and Genetic Stability of the Recombinant Duck Enteritis Viruses

**FIGURE 4:**
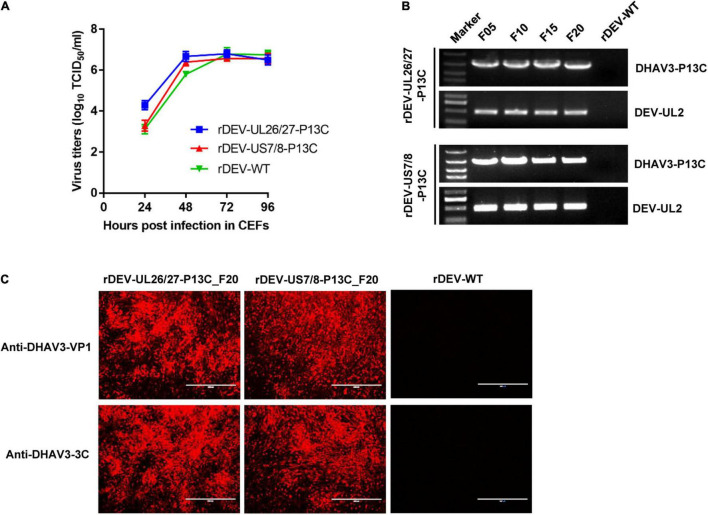
Growth kinetics and genetic stability of the recombinant DEVs in CEFs. **(A)** Comparison of the replication kinetics of the recombinant DEVs and the parental virus (rDEV-WT) in CEFs. **(B)** Detection of the P1 and 3C genes inserted in the recombinant DEVs passaged 5 (F05), 10 (F10), 15 (F15), and 20 (F20) times in CEFs. **(C)** Confirmation of P1 and 3C expression by the recombinant DEVs passaged 20 times in CEFs with immunofluorescence assay.

We tested whether insertion of the P1-P2A-3C cassette affected the *in vitro* replication of DEV. The CEF cultures infected with the viruses were harvested at different time points for titration in 96-well plates. The *in vitro* growth kinetics and magnitude of the two recombinant DEVs expressing DHAV-3 P1 and 3C were very similar to the parental virus rDEV-WT (Figure 4A), indicating that insertion of P1 and 3C did not affect the replication of the DEV vaccine strain.

To investigate whether the inserted P1 and 3C genes could be stably maintained in the recombinant viruses, we passaged the viruses in CEFs 20 times. The P1-P2A-3C cassette in both recombinants was detected by PCR amplification (Figure 4B). P1 and 3C protein expression by the serially passaged viruses was confirmed via immunofluorescence after 20 passages (Figure 4C).

### Antibody Responses Against DHAV-3 and Duck Enteritis Virus Induced by Recombinant Duck Enteritis Viruses in Ducks

**FIGURE 5:**
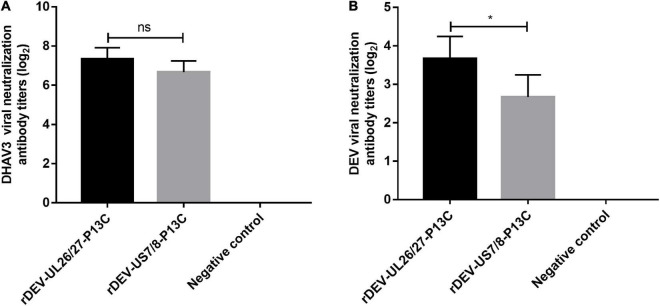
Antibody responses induced by the recombinant DEVs in ducks. Groups of twenty ducks were inoculated with 1000 ELD_50_ of the recombinant DEVs, and the sera samples were collected after 7 days of vaccination to detect neutralizing antibodies against DHAV-3 **(A)** and DEV **(B)**. The neutralizing antibody titers for ducks are expressed as log_2_. Data presented are the means ± standard deviations from twenty ducks per group. ns, no significant difference; *P < 0.05.

Groups of 20 ducks were inoculated with 1000 ELD_50_ of the recombinant viruses to evaluate the antibody responses against DHAV-3 and DEV induced by the recombinant DEVs. Anti-DHAV-3 neutralizing antibodies were detected from all ducks immunized with rDEV-UL26/27-P13C or rDEV-US7/8-P13C, as early as 7 days post-inoculation with mean titers of 7.3 and 6.7 (log_2_ values), respectively. The anti-DHAV-3 antibodies induced by these two viruses were comparable, with no significant difference (*P* > 0.05) (Figure 5A). The recombinant viruses rDEV-UL26/27-P13C and rDEV-US7/8-P13C also induced measurable anti-DEV neutralizing antibodies at 7 days post-inoculation, with mean titers of 3.7 and 2.6 (log_2_ values), respectively. The recombinant virus rDEV-UL26/27-P13C produced a higher level of neutralizing antibodies against DEV than rDEV-US7/8-P13C (*p* < 0.05) (Figure 5B).

### Protective Efficacy Against Lethal DHAV-3 and Duck Enteritis Virus Challenge in Ducks

**FIGURE 6:**
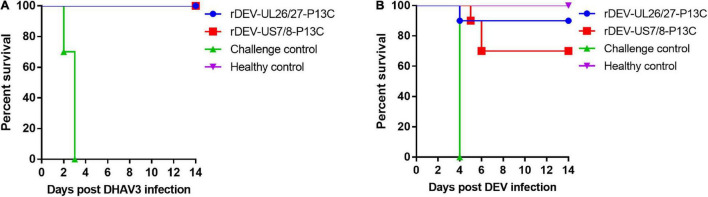
Protective efficacy of the recombinant DEVs against lethal DHAV-3 and DEV challenge in ducks. Ducks were inoculated with 1000 ELD_50_ of the recombinant DEVs and then challenged with 100 ELD_50_ of the virulent DHAV-3 A3 strain or 1000 minimum lethal doses of the virulent DEV CSC strain after 7 days of vaccination. **(A)** The survival rate of ducks challenged with DHAV-3 within an observation period of 14 days. **(B)** The survival rate of ducks challenged with DEV within an observation period of 14 days.

The ducks were inoculated with the recombinant viruses at 1 day of age and challenged with the virulent DHAV-3 A3 strain 7 days post-inoculation for protective efficacy evaluation. These ducks did not show any clinical signs in response to vaccination before the challenge. As shown in Figure 6A, the ducks in the rDEV-UL26/27-P13C and rDEV-US7/8-P13C vaccination groups were completely protected from lethal DHAV-3 challenge, showing no clinical signs of disease and no visible lesions in the liver, spleen, or other organs during the 2-week observation period. However, all ducks in the challenge control group showed severe clinical signs from 2 days post-challenge, including depression, lethargy, and anorexia; all these ducks died from the DHAV-3 challenge within 3 days. As expected, the ducks in the healthy control group did not show any clinical signs during the experiment. These results indicate that rDEV-UL26/27-P13C and rDEV-US7/8-P13C induced 100% protection against lethal DHAV-3 challenge in ducks.

We then evaluated the protective efficacy of the recombinant viruses against the DEV challenge. After the challenge, all the ducks in the challenge control group showed signs of disease, including listlessness, ruffled feathers, and anorexia, and died at 4 days post-challenge. Ninety percent (9/10) of the ducks in the rDEV-UL26/27-P13C group survived the DEV challenge and showed no clinical signs or gross lesions, indicating that rDEV-UL26/27-P13C vaccination provided 90% protection against DEV infection (Figure 6B). In comparison, vaccination with rDEV-US7/8-P13C conferred 70% (7/10) protection against DEV challenge. These results suggest that the recombinant viruses also induced solid protection against lethal DEV challenge in ducks.

## Discussion

Duck hepatitis A virus and DEV are highly prevalent pathogens in duck farms worldwide, resulting in more than 80% of mortality in ducklings (Chen et al., 2013; Li et al., 2013; Soliman et al., 2015; Lin et al., 2016). Vaccination provides the most effective protection against DHAV and DEV infection in ducks (Yugo et al., 2016; Dhama et al., 2017). The prevention of DVH mainly depends on the immunization of ducklings with commercial DHAV-1 live vaccines. However, the DHAV-1 vaccine failed to elicit cross-protection against DHAV-3, which currently severely affects Southeast Asia (Kim et al., 2009; Roan et al., 2015). Furthermore, simultaneous co-infection with DHAV and DEV has become increasingly prevalent in domestic duck farms in China and South Korea (Chen et al., 2013; Soliman et al., 2015). Thus, an effective and convenient bivalent vaccine against both DHAV-3 and DEV is highly recommended.

Live viral vector vaccines have been proven to effectively control avian diseases (Vannucc et al., 2013). Some recombinant vector vaccines have been granted licenses (Ge et al., 2007; DiNapoli et al., 2010). As a herpesvirus family member, DEV is a highly desirable live virus vector to generate multivalent vaccines. DEV-attenuated live vaccines rapidly induce protective immunity within several hours, and their efficacy appears to be unaffected by maternal antibodies (Liu et al., 2011a). DEV can establish latency after infection and induce long-term humoral and cellular immune responses (Dhama et al., 2017). The natural host range of DEV is limited to the order Anseriformes; hence, as a vaccine vector, it does not pose a risk to other domestic animals or humans. DEV has been widely applied to deliver protective antigens of avian pathogens, including avian influenza virus (Liu et al., 2011a; Sun et al., 2017), duck tembusu virus (Chen et al., 2014; Zou et al., 2016), and Newcastle disease virus (Ding et al., 2019). To address the urgent need for a single bivalent vaccine against both DEV and DHAV-3 infection, we generated two recombinant DEVs, with the P1 and 3C genes of a DHAV-3 virus inserted into the DEV vaccine strain genome.

To simultaneously express the three capsid proteins of DHAV-3 (VP0, VP1, and VP3), we cloned the P1 and 3C genes of DHAV-3 into a single expression vector and separated these genes using a self-cleaving 2A-element from porcine teschovirus-1 (Szymczak-Workman et al., 2012). Our immunofluorescence assay revealed that both P1 and 3C were successfully expressed in the cells infected with the recombinant virus. Since duck hepatitis is typically more lethal in younger ducklings, early vaccination of ducklings at hatching is important for preventing DHAV infection. In the present study, we evaluated the protective efficacy of recombinant DEVs against virulent DHAV-3 challenge in 1-day-old SPF ducks. After single-dose immunization, both rDEV-UL26/27-P13C and rDEV-US7/8-P13C elicited rapid immune responses against DHAV-3, as early as 7 days post-vaccination, conferring complete protection against DHAV-3 challenge. Moreover, the recombinant DEVs expressing DHAV-3 antigens provided solid protection against lethal DEV challenges. It appears that rDEV-UL26/27-P13C provided better protection against DEV challenge than rDEV-US7/8-P13C, suggesting that the UL26-UL27 gene junction site may be better than the US7-US8 site for use in developing DEV vector vaccines.

In summary, we developed recombinant DEV vector vaccines expressing the DHAV-3 P1 and 3C genes. These vaccines demonstrated good immunogenicity and provided rapid protection against lethal DHAV-3 and DEV challenges in ducks. The recombinant DEV-vectored DHAV-3 vaccine, which provides effective protection, holds great promise for use as a bivalent vaccine to combat DHAV-3 and DEV infection in ducks.

## Data Availability Statement

The datasets presented in this study can be found in online repositories. The names of the repository/repositories and accession number(s) can be found at: NCBI GenBank – KF263690.

## Ethics Statement

The animal study was reviewed and approved by the Animal Ethics Committee of Harbin Veterinary Research Institute of the Chinese Academy of Agricultural Sciences and performed in accordance with animal ethics guidelines and approved protocols [SYXK (Hei) 2017-009].

## Author Contributions

FY, KL, and YG conceived and designed the experiments. FY and KL performed the experiments and wrote the manuscript. PL, XL, RL, KL, YG, and LG analyzed the data. HC, YZ, CL, XQ, QP, AL, and XW contributed reagents, materials, and analysis tools. All authors read and approved the final manuscript.

## Conflict of Interest

The authors declare that the research was conducted in the absence of any commercial or financial relationships that could be construed as a potential conflict of interest.

## Publisher’s Note

All claims expressed in this article are solely those of the authors and do not necessarily represent those of their affiliated organizations, or those of the publisher, the editors and the reviewers. Any product that may be evaluated in this article, or claim that may be made by its manufacturer, is not guaranteed or endorsed by the publisher.
